# The impact of negative events on adolescent sleep quality: the role of negative attention bias and negative emotions

**DOI:** 10.3389/fpsyg.2026.1734257

**Published:** 2026-04-07

**Authors:** Tingjun Yong, Haining Wang, Yue Cai, Jingjing Huo, Wenbo Wei, Yonghui Wang, Chao Fu

**Affiliations:** 1 School of Psychology, Shaanxi Normal University, Xi'an, Shaanxi, China; 2 School of Education, Qinghai Normal University, Xining, Qinghai, China; 3 School of Law and Sociology, Qinghai Normal University, Xining, Qinghai, China; 4 Qinghai Provincial Psychological Association, Xining, Qinghai, China; 5 Research Institute of Plateau Science and Sustainable Development of Qinghai Normal University, Xining, Qinghai, China; 6 School of Music, Qinghai Normal University, Xining, Qinghai, China; 7 School of Educational Science, Guangxi Minzu Normal University, Chongzuo, Guangxi, China; 8 State Grid Qinghai Electric Power Company, Xining Power Supply Company, Xining, Qinghai, China

**Keywords:** anxiety, negative attention bias, negative events, sleep quality, undergraduate student

## Abstract

**Background:**

Sleep is a critical factor affecting college students’ physical and mental health, closely intertwined with their daily lives and academic performance. While previous studies have examined the relationship between negative life events and sleep quality among college students, few have explored the mediating role of negative attentional bias and negative emotions in this relationship. Based on cognitive-behavioral theory, this study investigates the impact of negative life events on college students’ sleep quality and examines the mediating roles of negative attentional bias and anxiety.

**Methods:**

A total of 566 college students were assessed using the Adolescent Life Events Scale, Negative Attention Bias Scale, Depression-Anxiety-Stress Scale, and Pittsburgh Sleep Quality Index.

**Results:**

Negative attention bias and anxiety mediate the relationship between negative life events and sleep quality, acting as independent mediators and chain mediators, respectively. Negative events exert a significant positive influence on poor sleep quality (*β* = 0.181, *p* < 0.001), negative events exert a significant positive influence on negative attentional bias (*β* = 0.355, *p* < 0.001) and anxiety (*β* = 0.306, *p* < 0.001). Negative attention positively influenced anxiety (*β* = 0.196, *p* < 0.001) and poorer sleep quality (*β* = 0.115, *p* < 0.01). Anxiety also significantly positively influenced poorer sleep quality (*β* = 0.658, *p* < 0.001).

**Conclusion:**

These findings contribute to understanding the mechanisms underlying sleep quality in college students and provide insights for sleep quality interventions.

## 1 Introduction

Sleep health is a critical factor influencing college students’ physical and mental well-being. It not only significantly impacts their daily lives and academic performance but is also closely associated with psychological issues such as depression and anxiety (Curcio et al., 2006; Demirci et al., 2015). The China Sleep Research Report (2025) indicates that over 65% of respondents have experienced sleep disturbances. The Healthy China Initiative (2019–2030) has incorporated sleep interventions into its “Mental Health Promotion Action,” further highlighting sleep health’s critical role within the public health system. Amid intensifying competition in higher education and mounting employment pressures, college students face increasingly severe sleep health challenges. Sleep quality issues driven by psychological, behavioral, and environmental factors are prevalent among this demographic, emerging as a critical public health concern (Kong et al., 2025). This study is grounded in cognitive-behavioral theory, which posits that cognition, emotion, and behavior interact with one another. Among these, an individual’s cognitive appraisal of events serves as a key factor influencing both emotion and behavior (Beck, 1979). Sleep quality issues can be regarded as behavioral manifestations of specific cognitive-emotional processes. Consequently, this study aims to explore the determinants of sleep quality among college students, enriching research on the mechanisms influencing sleep quality and providing a theoretical foundation for scientific prevention and intervention strategies.

### 1.1 Negative events and poorer sleep quality among college students

Negative events refer to experiences or environments encountered by individuals that exceed their coping capacity and pose a threat to their physical and mental health. Such events typically trigger negative emotions in individuals (Skaggs and Barron, 2006). Stress response theory posits that negative events, as typical stressors, activate the hypothalamic–pituitary–adrenal (HPA) axis and sympathetic nervous system, triggering cognitive arousal such as rumination and worry, which subsequently disrupts sleep architecture (Buckley and Schatzberg, 2005; Lupien et al., 2009). Furthermore, negative events serve as significant risk factors for mental health issues such as psychological distress, anxiety, and depression, potentially increasing individuals’ susceptibility to other psychosomatic disorders (Luecken and Gress, 2010; Marum et al., 2014). As the severity of negative event stress increases, the resulting stress responses—such as heightened vigilance—may impair an individual’s sleep quality (Ren et al., 2021; Baddam et al., 2019). Therefore, this study proposes,

*Hypothesis* 1: Negative events are positively correlated with poor sleep quality among college students.

### 1.2 The mediating role of negative attention bias

Negative attentional bias refers to an individual’s tendency to prioritize processing or pay greater attention to negative information in the environment compared to neutral or positive information (Smith et al., 2006). Previous research indicates that negative life events experienced by individuals, particularly those occurring early in life or repeatedly, are associated with long-term dysregulation in cognitive processes. Specifically, individuals may spend more time regulating negative emotions or exhibit attentional bias toward threatening information, leading to faster responses to negative stimuli (Shackman et al., 2007). Harvey (2002) cognitive model of insomnia maintenance posits that negative attentional bias toward internal or external threat cues triggers autonomic arousal and emotional distress. This sustained monitoring state may impair sleep quality. Empirical research indicates a significant positive correlation between individuals’ attentional bias toward threatening information and sleep quality issues, though these findings are limited to the specific developmental stage of adolescence (Ricketts et al., 2018). Therefore, this study proposes,

*Hypothesis* 2: Negative attention bias mediates the relationship between negative events and sleep quality.

### 1.3 The mediating role of anxiety

Negative emotions are characterized by an individual’s subjective experience of unpleasantness, such as fear, disgust, anxiety, and other emotional states; among these, anxiety ranks as one of the most prevalent negative emotions among college students (Watson et al., 1988; Kavvadas et al., 2023). Anxiety represents an individual’s persistent state of heightened vigilance toward potential threats, fundamentally marked by excessive autonomic nervous system activation and accompanying subjective feelings of tension and discomfort (Lovibond and Lovibond, 1995). Stress and coping theories suggest that negative events experienced by individuals trigger emotional responses through cognitive evaluation processes; when individuals evaluate events as threatening, they are more likely to experience negative emotions such as anxiety (Biggs et al., 2017). Empirical research indicates that emotional issues such as anxiety and depression are closely associated with sleep quality problems (Alvaro et al., 2013; Nyer et al., 2013), and that excessive worry accompanying anxiety reduces sleep quality (Harvey, 2002). Therefore, this study proposes,

*Hypothesis* 3: Anxiety mediates the relationship between negative events and sleep quality.

In summary, both negative attentional bias and anxiety may serve as important mediating variables between negative events and sleep quality, and the two are also interconnected. Empirical research findings indicate that negative attentional bias is significantly correlated with depression and anxiety, inducing negative emotions in individuals (Li and Li, 2022). Negative attentional bias amplifies individuals’ emotional responses to negative events, leading to short-term fluctuations in anxiety and thereby increasing anxiety susceptibility (Iijima et al., 2018; MacLeod et al., 2019). Research indicates a significant positive correlation between anxiety and sleep quality (Demirci et al., 2015). Based on cognitive-behavioral theory, individuals who experience negative events are more prone to developing negative cognitive schemas, generating negative attentional bias toward threatening information, and subsequently triggering anxiety. The physiological arousal and subjective discomfort resulting from anxiety disrupt sleep architecture, ultimately impairing sleep quality (Beck, 1979). Therefore, this study proposes,

*Hypothesis* 4: Negative attentional bias and anxiety may exert a chain-mediated effect between negative life events and sleep quality.

**Figure 1:**
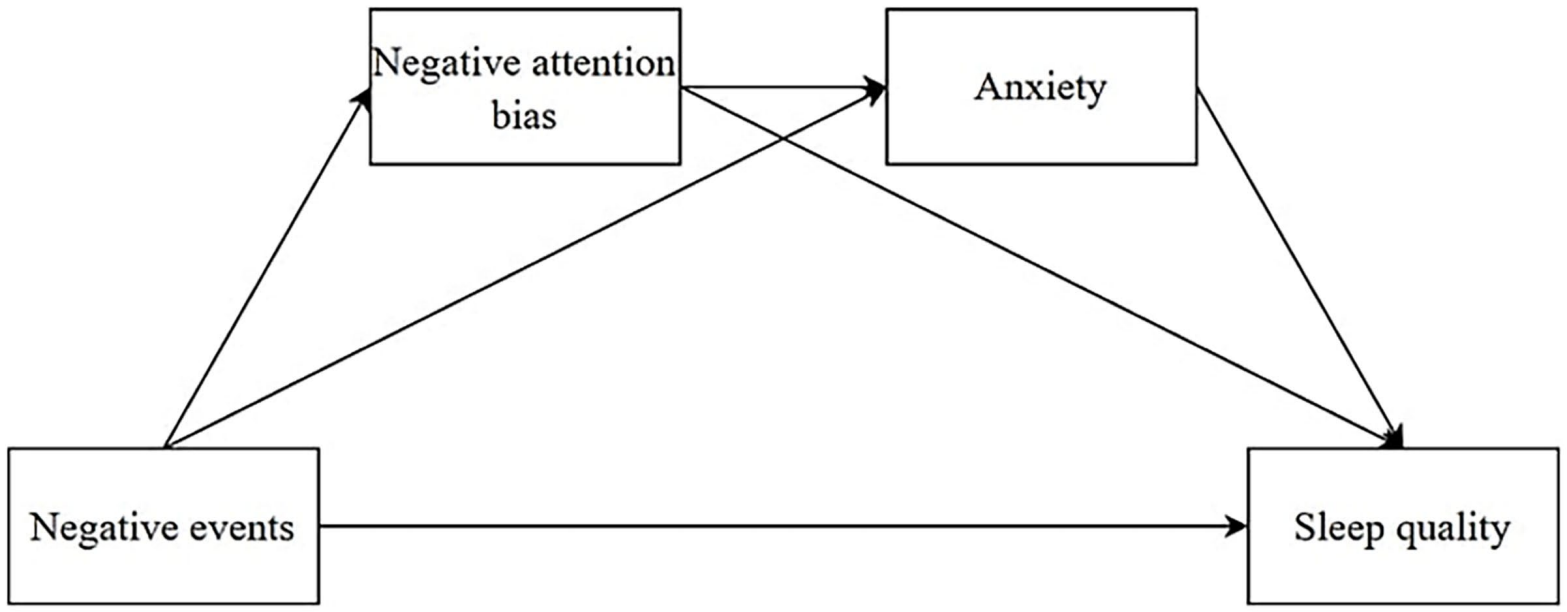
Research hypothesis model diagram.

In summary, this study aims to establish a chain mediation model to examine the mechanism through which negative events affect college students’ sleep quality, exploring the mediating roles of negative attention bias and anxiety in this process. This approach offers new perspectives and evidence for sleep quality research. The hypothesized model is illustrated in Figure 1.

## 2 Methods

### 2.1 Participants

Using G*Power 3.1 for a pre-test analysis, we selected multiple regression analysis with a medium effect size f^2^ = 0.15, significance level *α* = 0.05, and statistical power 1-*β* = 0.95. This yielded a minimum required sample size of 119. Cluster sampling was employed, targeting university students from a school in Qinghai Province. Prior to the survey, researchers clearly explained the study objectives. Questionnaires were administered anonymously to protect personal privacy. After ensuring informed consent from participants, survey links were distributed via the Chinese online survey platform “WJX,” resulting in 590 completed questionnaires.

Invalid questionnaires were excluded based on the following criteria: incorrect responses to the lie-detection question, consecutive selections of the same option within a single questionnaire, or completion times that were excessively short (<4 min) or long (>30 min). A lie-detection question was inserted into the survey: “Please select ‘Yes’ if you are answering seriously.” Incorrect responses to this question were deemed indicative of non-serious completion and resulted in exclusion (Zhang et al., 2025).

Ultimately, 566 valid questionnaires were obtained, yielding a valid response rate of 96%. Participants comprised 147 males (26%) and 419 females (74%), with 149 freshmen (26%), 204 sophomores (36%), and 213 juniors (38%). This study received approval from the Ethics Committee of Qinghai Normal University (Approval No.: QHNU2025LS-03).

### 2.2 Measurement tools

#### 2.2.1 Adolescent self-rating life events check list

The Youth Life Events Scale developed by Liu et al. (1997) and revised by Xin and Yao (2015) was employed, comprising 26 items such as “being misunderstood or wrongly blamed” and “experiencing discrimination or cold treatment.” A 5-point rating scale was used: 1 = did not occur or had no impact, 2 = mild impact, 3 = moderate impact, 4 = severe impact, 5 = extremely severe impact. A higher total score indicates a greater impact from negative life events experienced within the past year. In this study, the Cronbach’s *α* coefficient for this scale was 0.96. The KMO coefficient is 0.96.

#### 2.2.2 Negative attention bias scale

The Negative Attention Bias Scale, validated by Noguchi et al. (2006) and revised by Lv et al. (2016), comprises 11 items such as “I worry that bad things will happen to me” and “Sometimes I think others want to deceive me.” It employs a 5-point Likert scale, where 1 = strongly disagree and 5 = strongly agree. Higher total scores indicate stronger negative attention bias in individuals. In this study, the Cronbach’s *α* coefficient for this scale was 0.93. The KMO coefficient is 0.94.

#### 2.2.3 Depression-anxiety-stress scale

The anxiety subscale from the Chinese version of the Depression-Anxiety-Stress Scale, developed by Lovibond and Lovibond (1995) and revised by Gong et al. (2010), was employed. This subscale comprises seven items, such as “I feel trembling” and “I feel like I’m about to panic.” Items were rated on a 4-point Likert scale (1 = Not at all true, 4 = Always true). Higher scores on a dimension indicate greater severity of corresponding emotional symptoms. In this study, the anxiety subscale achieved a Cronbach’s α coefficient of 0.90. The KMO coefficient is 0.92.

#### 2.2.4 Pittsburgh sleep quality index

The Pittsburgh Sleep Quality Index (PSQI), developed by Buysse et al. (1989) and revised by Liu et al. (1996), comprises 19 self-rated and 5 other-rated items. Among these, 18 self-rated items are scored to form seven components. Using a 4-point scale, the total score ranges from 0 to 21, with higher scores indicating poorer sleep quality. In this study, the Cronbach’s α coefficient for the seven components was 0.75. The KMO coefficient is 0.92.

### 2.3 Data processing and analysis

Data analysis was conducted using SPSS 27.0, Amos 29.0, and Mplus 8.3. Descriptive statistics and correlation analyses were performed with SPSS 27.0, while confirmatory factor analysis was conducted using Amos 29.0. To examine the mediating roles of negative attention bias and anxiety in the relationship between negative events and poor sleep quality, Mplus 8.3 was employed. Parameter estimation was based on bootstrap sampling with 5,000 resamples, and a 95% confidence interval excluding zero was considered statistically significant.

## 3 Results

### 3.1 Confirmatory factor analysis

This study employed Amos 29.0 to conduct confirmatory factor analysis on four variables: negative events, negative attention bias, anxiety, and sleep quality. As shown in Table 1, the four-factor model exhibited the best fit indices (*χ^2^*/*df* = 3.172, CFI = 0.811, TLI = 0.804, RMSEA = 0.062), indicating that the discriminant validity among the four variables was relatively superior to other models, representing four distinct constructs.

**Table 1:** Results of confirmatory factor analysis (*N* = 566).

Model	Combination	*χ^2^*/*df*	CFI	TLI	RMSEA
Four-factor	A, B, C, D	3.172	0.811	0.804	0.062
Three-factor	A, B, C + D	3.805	0.755	0.738	0.070
Two-factor	A, B + C + D	5.052	0.645	0.620	0.085
Single-factor	A + B + C + D	6.954	0.482	0.464	0.103

A denotes negative events, B denotes negative attentional bias, C denotes anxiety, D denotes sleep quality; + denotes factor combination.

### 3.2 Common method bias test

The Harman single-factor test for common method bias revealed 10 factors with eigenvalues exceeding 1. The first factor explained 28.54% of the variance, falling below the 40% critical threshold (Zhou and Long, 2004). Therefore, no significant common method bias was identified in this study.

### 3.3 Correlation analysis of negative events, negative attention bias, anxiety, and sleep quality

Correlation analysis of the total mean scores for negative events, negative attention bias, anxiety, and sleep quality revealed significant positive correlations between negative events and each of the other three variables (see Table 2). Sleep quality showed a significant positive correlation with negative events (*r* = 0.40, *p* < 0.001); negative attentional bias exhibited a significant positive correlation with anxiety (*r* = 0.32, *p* < 0.001); simultaneously, negative life events showed significant positive correlations with both negative attentional bias (*r* = 0.33, *p* < 0.001) and anxiety (*r* = 0.40, *p* < 0.001). Referencing previous studies, variables such as gender were not controlled for Zheng et al. (2025). To validate the robustness of the findings, a sensitivity analysis was conducted by rerunning Model 6 with gender as a covariate. Results indicate no substantial changes in the estimated values, Bootstrapped 95% confidence intervals, or significance levels across all mediating paths. This demonstrates that the primary findings of this study are insensitive to the covariate of gender and are robust.

**Table 2:** Correlation analysis of each variable.

Variable	*M*	*SD*	1	2	3	4
1 Negative events	1.66	0.64	1			
2 Negative attentional bias	2.78	0.83	0.33^***^	1		
3 Anxiety	1.70	0.63	0.40^***^	0.32^***^	1	
4 Sleep quality	8.40	3.52	0.40^***^	0.37^***^	0.61^***^	1

^*^p<0.05, ^**^p<0.01, ^***^p<0.001.

### 3.4 Relationship between negative events and sleep quality: testing the chain mediation model

**Figure 2:**
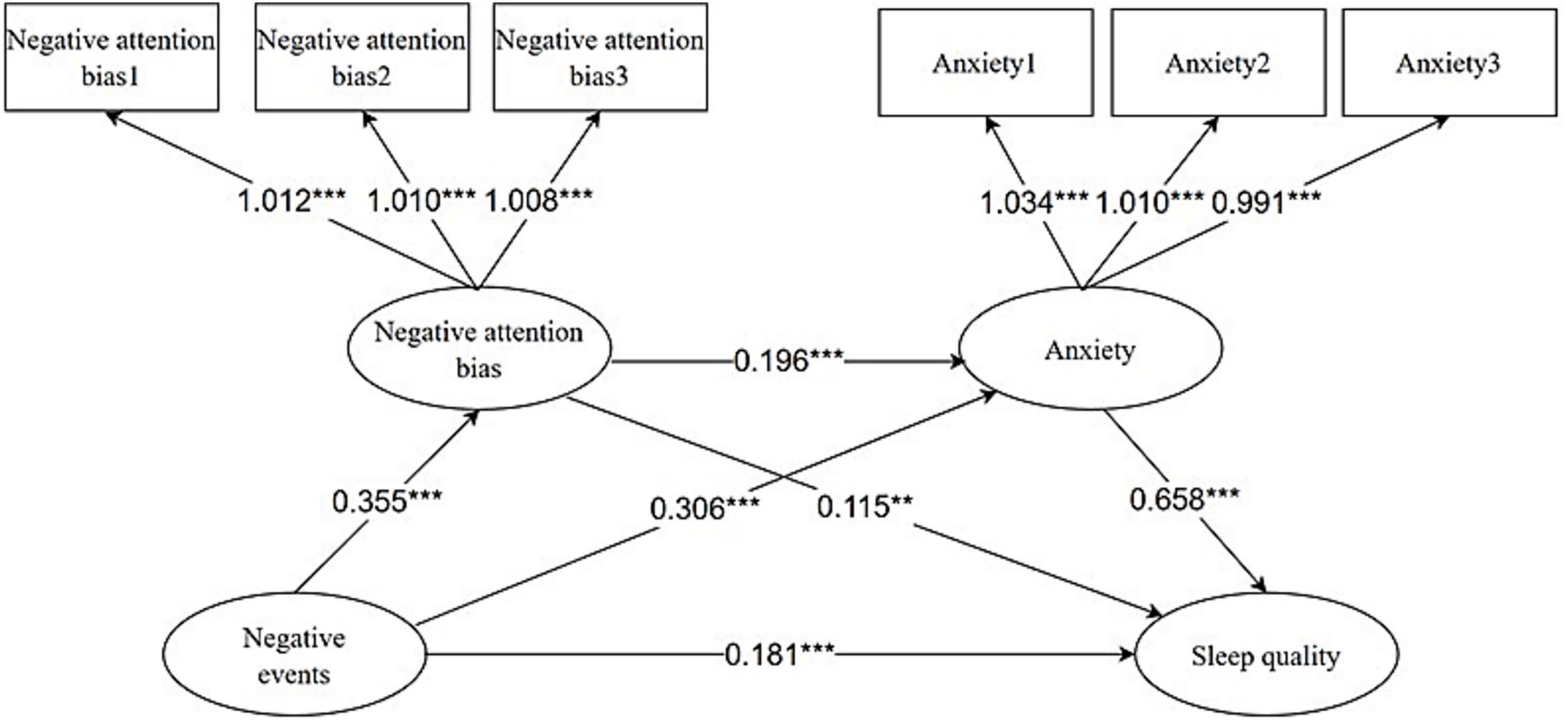
Negative attention bias and anxiety chain mediation model. **p < 0.01, ***p < 0.001.

The Negative Attention Bias and Anxiety scales used in this study are unidimensional but contain numerous items. To address the substantial parameter demands in modeling, items were bundled into three indicators using the Item-Structure Equilibrium method (Little et al., 2002). We then tested a chained mediation model within a structural equation modeling framework, positioning negative events as the independent variable, negative attentional bias and anxiety as sequential mediators, and sleep quality as the dependent variable. Model fit was assessed based on the criteria established in prior research (Chen et al., 2018), in which a *χ^2^*/*df* ratio < 3, CFI and TLI values > 0.8, and an RMSEA < 0.08 were considered indicative of acceptable fit. The model demonstrated acceptable fit indices (*χ^2^*/*df* = 2.94, CFI = 0.834, TLI = 0.826, RMSEA = 0.058). As shown in Table 3 and Figure 2, negative events exert a positive influence on poorer sleep quality (*β* = 0.181, *p* < 0.001). Negative events also exert a significant positive influence on negative attentional bias (*β* = 0.355, *p* < 0.001) and anxiety (*β* = 0.306, *p* < 0.001). Negative attention positively influenced anxiety (*β* = 0.196, *p* < 0.001) and poorer sleep quality (*β* = 0.115, *p* < 0.01). Anxiety also significantly positively influenced poorer sleep quality (*β* = 0.658, *p* < 0.001).

**Table 3:** Test of the mediating effects of negative attentional bias and anxiety.

Variable	Negative attentional bias			Anxiety			Sleep quality		
	*β*	*SE*	*t*	*β*	*SE*	*t*	*β*	*SE*	*t*
Negative events	0.355	0.041	8.583^***^	0.306	0.039	7.835^***^	0.181	0.049	3.677^***^
Negative attentional bias				0.196	0.036	5.442^***^	0.115	0.040	2.871^**^
Anxiety							0.658	0.043	15.269^***^
*R^2^*		0.029			0.028			0.032	

^*^p<0.05, ^**^p<0.01, ^***^p<0.001.

As shown in Table 4, the direct effect of negative events on poor sleep quality among college students was significant (effect size = 0.299, 95% CI [0.601, 0.955]). The total indirect effect of negative attentional bias and anxiety was significant (effect size = 0.473, 95% CI [0.361, 0.592]), accounting for 61.27% of the total effect. Among these, the mediating effect of negative attention bias was significant (effect size = 0.067, 95% CI [0.024, 0.122], accounting for 8.68%). The mediating effect of anxiety was significant (effect size = 0.331, 95% CI [0.219, 0.440], accounting for 42.88%). The chain-mediated effect of negative attention bias on anxiety was equally significant (effect size = 0.075, 95% CI [0.045, 0.113], accounting for 9.71%).

**Table 4:** Proportion of effects in the serial mediation model of negative attentional bias and anxiety.

Effect path	Effect size	Boot SE	95% Confidence interval		Proportion
			LL	UL	
Total effect	0.772	0.095	0.601	0.955	
Direct effect	0.299	0.083	0.090	0.283	
Total Indirect Effect	0.473	0.060	0.361	0.592	61.27%
Negative events → Negative attentional bias →Sleep quality	0.067	0.025	0.024	0.122	8.68%
Negative events → Anxiety →Sleep quality	0.331	0.056	0.219	0.440	42.88%
Negative events → Negative attentional bias → Anxiety →Sleep quality	0.075	0.017	0.045	0.113	9.71%

## 4 Discussion

This study, grounded in cognitive-behavioral theory, examines the impact of negative events on college students’ sleep quality. By introducing negative attentional bias and anxiety as mediating variables, it partially elucidates the relationship between negative events and sleep quality, along with its underlying mechanisms. The findings hold significant theoretical and practical value for the scientific prevention and intervention of sleep quality issues among college students.

The findings of this study indicate that negative events are positively correlated with poorer sleep quality, validating Hypothesis 1. This aligns with previous research findings (Ren et al., 2021; Baddam et al., 2019). The persistent accumulation of negative events is closely associated with individual sleep problems. This process involves dysfunction of the hypothalamic–pituitary–adrenal (HPA) axis, and the anterior insula significantly amplifies the stress response triggered by these events (Kim et al., 2022). Stress response theory posits that negative events—perceived as threatening and challenging experiences—impact sleep architecture through physiological and cognitive arousal (Buckley and Schatzberg, 2005; Lupien et al., 2009). These findings support the stress response theory, indicating that negative events as stressors can lead to sleep problems in individuals. Therefore, early intervention can be implemented for individuals who have experienced negative events. It is worth noting that the negative impact of adverse events on sleep quality may also be moderated by individual factors. Individuals adept at cognitive reappraisal may alter their perceptions of negative events, suggesting that cognitive reappraisal may moderate the early pathway of the model (Ye et al., 2018).

In this study, negative attentional bias was found to be associated with poorer sleep quality and partially mediated the relationship between negative events and sleep disturbances, thereby validating, Hypothesis 2. The cognitive model of insomnia maintenance suggests that individuals’ attentional bias toward threatening cues is associated with sleep quality issues (Harvey, 2002). Individuals who experience negative events are prone to develop attentional biases toward threatening information, leading to faster responses to negative stimuli (Shackman et al., 2007). Consistent with previous research, this study found a significant correlation between negative attentional bias and sleep quality; that is, individuals’ focus on threatening cues induces heightened cognitive arousal, thereby disrupting sleep (Ricketts et al., 2018). The findings indicate that negative attentional bias mediates the relationship between negative life events and sleep quality, revealing that individuals who have experienced negative events may develop sleep quality issues through this cognitive pattern of negative attentional bias.

This study found that anxiety partially mediated the relationship between negative events and poorer sleep quality, validating Hypothesis 3. Stress and coping theory posits that when individuals evaluate negative events as threatening, it significantly increases the likelihood of negative emotional responses such as anxiety. That is, individuals who experience negative events cognitively transform them into anxiety (Biggs et al., 2017). Anxiety represents a state of persistent heightened vigilance toward potential threats. Its excessive physiological arousal and subjective discomfort disrupt sleep architecture, ultimately leading to sleep disturbances (Yong, 2017; Silva et al., 2020). These findings highlight anxiety’s mediating role between negative events and sleep quality, supporting and deepening the stress-coping theory. They demonstrate that anxiety serves as a crucial mechanism through which stress influences sleep.

This study also found that negative attention bias and anxiety exerted a chain-mediated effect in the association between negative events and poorer sleep quality among college students, thereby validating Hypothesis 4. Negative attentional bias amplifies individuals’ emotional responses to negative events, triggering short-term anxiety fluctuations and thereby increasing their susceptibility to anxiety (Iijima et al., 2018; MacLeod et al., 2019). Individuals experiencing negative events face heightened mental health risks, making them more prone to developing negative cognitive schemas and attention biases toward threatening information, which in turn generates anxiety. Anxiety-related physiological hyperarousal and rumination disrupt sleep architecture, leading to sleep quality issues (Beck, 1979; Luecken and Gress, 2010; Marum et al., 2014).

This study examined the chained mediation hypothesis of “negative events → negative attentional bias → anxiety → sleep quality,” providing preliminary and exploratory empirical support for this pathway. It holds both theoretical and practical significance. Theoretically, the introduction of negative attentional bias and anxiety provides deeper insights into how negative events impact sleep quality, thereby expanding the cognitive-behavioral model. Practically, these findings suggest that university mental health professionals can mitigate sleep quality issues among college students by implementing sleep health education and cognitive-behavioral interventions. This approach helps block the effects of negative events, enabling scientific prevention and targeted interventions to improve student sleep quality. For instance, regarding the pathway “negative events → negative attention bias,” university mental health professionals can establish routine psychological stress screening mechanisms, paying particular attention to students’ mental well-being during critical periods such as the start of the semester and exam weeks. Addressing the pathway “negative attention bias → anxiety,” university mental health courses can emphasize teaching cognitive restructuring techniques within cognitive behavioral therapy.

This study also has the following limitations: First, the sample originates from a single university, so caution is warranted when generalizing the findings. Future research should expand the sampling scope and replicate the findings across a broader undergraduate population. Second, while the cross-sectional model developed in this study is grounded in theoretical assumptions, the causal relationships among variables require further validation through longitudinal studies or experimental interventions. Participants may be required to report daily negative events and sleep quality from the previous night for several consecutive weeks (Jiang et al., 2023), or interventions may employ cognitive restructuring techniques to address negative thought patterns arising when individuals cope with adverse events (Zhao, 2021). Future research may employ either approach to test the chained mediation model established here and clarify the causal dynamics between variables.

## Data Availability

The raw data supporting the conclusions of this article will be made available by the authors, without undue reservation.
