# Correction: The impact of negative events on adolescent sleep quality: the role of negative attention bias and negative emotions

**DOI:** 10.3389/fpsyg.2026.1852382

**Published:** 2026-05-14

**Authors:** Tingjun Yong, Haining Wang, Yue Cai, Jingjing Huo, Wenbo Wei, Yonghui Wang, Chao Fu

**Affiliations:** 1 School of Psychology, Shaanxi Normal University, Xi'an, Shanxi, China; 2 School of Education, Qinghai Normal University, Xining, Qinghai, China; 3 School of Law and Sociology, Qinghai Normal University, Xining, Qinghai, China; 4 Qinghai Provincial Psychological Association, Xining, Qinghai, China; 5 Research Institute of Plateau Science and Sustainable Development of Qinghai Normal University, Xining, Qinghai, China; 6 School of Music, Qinghai Normal University, Xining, Qinghai, China; 7 School of Educational Science, Guangxi Minzu Normal University, Chongzuo, Guangxi, China; 8 State Grid Qinghai Electric Power Company, Xining Power Supply Company, Xining, Qinghai, China

**Keywords:** anxiety, negative attention bias, negative events, sleep quality, undergraduate student

In the published article, the affiliation “Research Institute of Plateau Science and Sustainable Development of Qinghai Normal University, Xining, Qinghai, China” was erroneously assigned to author Yue Cai. They are affiliated with affiliations 4 and 6.

There was a mistake in the caption of Figure 2 as published. The significance levels were incorrectly given as “^***^p < 0.01, ^**^p < 0.05”. The corrected caption of Figure 2 appears below.

“Figure 2. Negative attention bias and anxiety chain mediation model. ^**^p < 0.01, ^***^p < 0.001.”

The original version of this article has been updated.

